# Liquid-crystalline calcium carbonate: biomimetic synthesis and alignment of nanorod calcite†
†Electronic supplementary information (ESI) available. See DOI: 10.1039/c5sc01820j


**DOI:** 10.1039/c5sc01820j

**Published:** 2015-08-12

**Authors:** Masanari Nakayama, Satoshi Kajiyama, Tatsuya Nishimura, Takashi Kato

**Affiliations:** a Department of Chemistry and Biotechnology , School of Engineering , The University of Tokyo , Hongo, Bunkyo-ku , Tokyo 113-8656 , Japan . Email: kato@chiral.t.u-tokyo.ac.jp

## Abstract

Liquid-crystalline CaCO_3_ has been prepared for the first time. The nanorods of CaCO_3_ calcite are obtained by bio-inspired crystallization through aqueous colloidal precursors of amorphous CaCO_3_ stabilized by poly(acrylic acid). The synthesized calcite nanocrystals have well-tuned morphologies that are preferable for formation of liquid-crystalline phases in concentrated aqueous colloidal solution. The one-dimensional alignment of calcite crystals is achieved by mechanical shearing of the aqueous colloidal solution showing liquid-crystalline phases. These CaCO_3_-based liquid crystals formed by a self-organization process in mild conditions may have great potential for use as environmentally friendly materials.

## Abstract 2

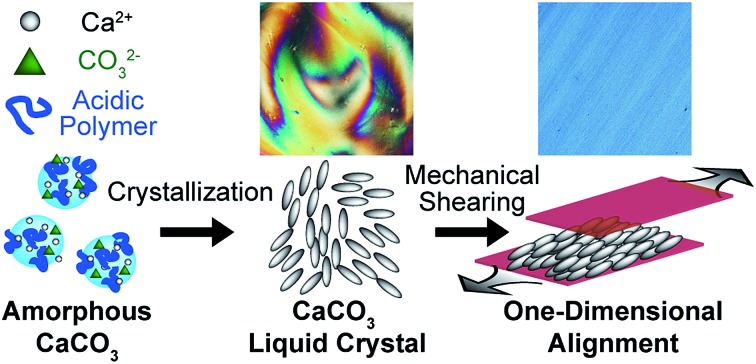
Liquid-crystalline CaCO_3_ crystals were obtained by bio-inspired crystallization through amorphous CaCO_3_. These calcite nanorods were macroscopically aligned by applying mechanical force to the liquid-crystalline phase.

## Introduction

In nature, living organisms produce assembled and highly oriented structures composed of polymers and inorganic nanoparticles.^1^–^5^ These oriented structures play significant roles in the exhibition of a variety of functions.^1^–^6^ For example, in mollusk shells, rod-shaped CaCO_3_ crystals are aligned to form 2D arrays^4^ and for sea urchin teeth, calcite needles and plates are co-aligned.^5^ In bones and teeth one-dimensionally aligned nanocrystals of calcium phosphate form hierarchically assembled structures.^6^ These biominerals show high mechanical strength. The relationships between structures and functions have been investigated.^1^–^6^ But the formation processes of these biominerals are yet to be sufficiently revealed.

Our intention is to develop synthetic liquid-crystalline CaCO_3_ calcite to obtain assembled and ordered inorganic crystals. Liquid-crystalline states take advantage of alignment control for highly organized structures.^7^–^9^ We expected that if liquid-crystalline CaCO_3_ can be synthesized and processed using the liquid-crystalline state, novel optically functional and mechanically tough materials based on oriented inorganic crystals may be developed.

It was reported that colloidal suspensions of anisotropic organic and inorganic nano- and micro-objects show liquid-crystalline behavior.^10^ For example, polysaccharide fibers,10b,c clay nanosheets,10*d* gibbsite plate-like particles10*e* and silica rod-like particles10*f* have been studied as colloidal liquid crystals. These liquid-crystalline materials can lead to new functions, such as anisotropic photoelectric and photocatalytic properties.10*g* However, as far as we know liquid-crystalline CaCO_3_ has not yet been obtained.

Our strategy was to use an amorphous calcium carbonate (ACC) phase as a precursor to synthesize liquid-crystalline CaCO_3_ crystals. Crystallization through amorphous precursors stabilized by acidic polymers is considered to be key for the precise morphological control of CaCO_3_ crystals.3b,c,^11^ Morphological control of CaCO_3_ by solution-based syntheses has been widely studied in the presence of molecular additives.3b,c,11a,b,^12^

**Fig. 1:**
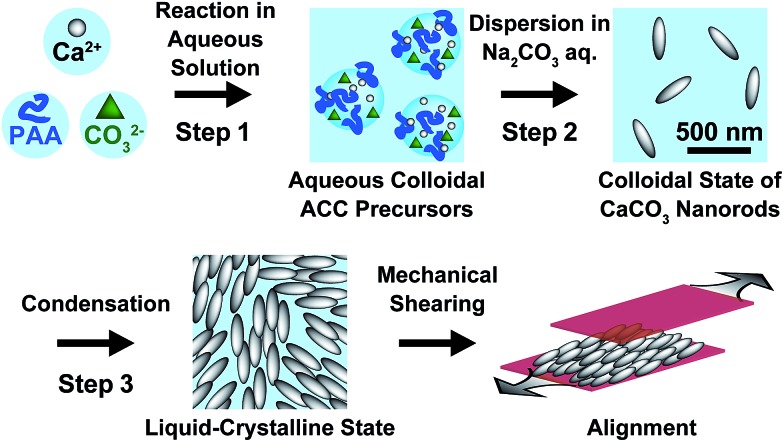
Synthetic approach and alignment control for liquid-crystalline CaCO_3_.

Here we report on liquid-crystalline CaCO_3_ calcite. Calcite nanocrystals with rod-shaped structures were formed through ACC precursors stabilized by poly(acrylic acid) (PAA) (Fig. 1).

## Results and discussion

**Fig. 2:**
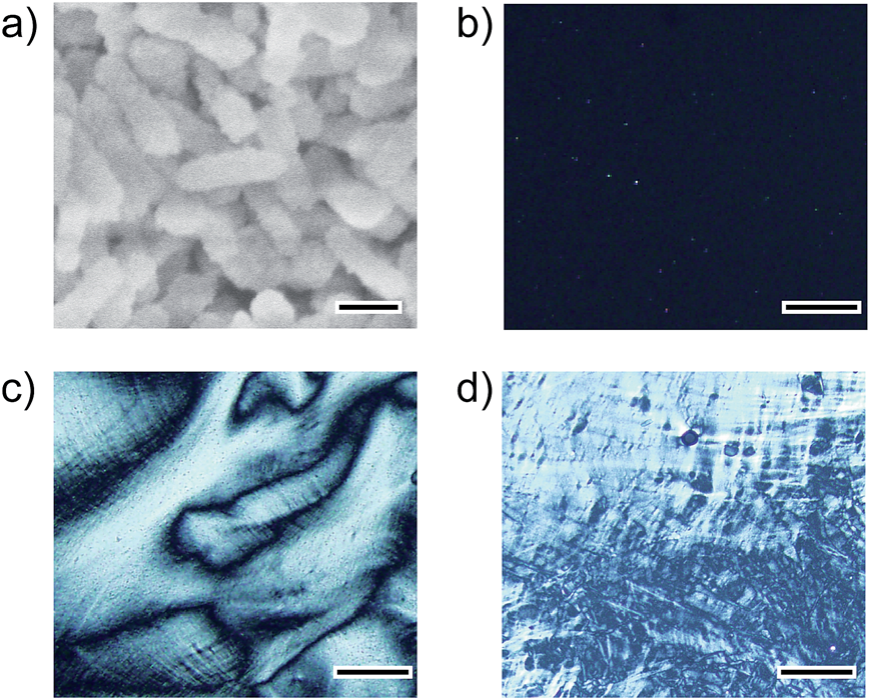
(a) SEM image of calcite nanocrystals (bar: 200 nm). (b–d) Phase behavior of calcite nanocrystal aqueous suspensions at different volume fractions (bar: 100 μm); (b) 14.7 vol% (isotropic phase), (c) 31.2 vol% (liquid-crystalline phase), (d) 32.5 vol% (phase separation of the liquid-crystalline phase and aggregated particles).

Rod-shaped CaCO_3_ nanocrystals were successfully prepared using ACC precursors obtained as colloidal suspensions in the presence of 7.2 × 10^–1^ wt% PAA (Fig. 2). The procedure for the preparation of ACC was reported in our previous paper (Step 1 in Fig. 1).^13^ The amorphous colloidal precursors were concentrated by centrifugation. Then, the precursors were re-dispersed in 25 mM Na_2_CO_3_ aqueous solution for crystallization (Step 2 in Fig. 1). During the process, the turbidity of the reaction solution increased, which suggests that transformation from the amorphous precursors to crystalline CaCO_3_ particles occurred within 72 h of stirring. The resultant CaCO_3_ particles exhibited a rod-shaped morphology at a submicrometer scale as observed by scanning electron microscopy (SEM) (Fig. 2a). The crystalline phase of the nanocrystals was identified as calcite by X-ray diffraction (XRD) measurements and Fourier-transform infrared (FTIR) spectroscopy (see Fig. S1 and S2 in the ESI†).

We found that the aqueous colloidal solution of calcite nanocrystals showed liquid-crystalline phases (Step 3 in Fig. 1). Polarizing optical microscope (POM) images of the calcite nanocrystal suspensions are shown in Fig. 2b–d. A dark image of the isotropic phase at 15 vol% of calcite nanocrystals is seen in Fig. 2b. As the suspension is concentrated to higher than 23 vol%, a birefringent texture is observed with POM, which suggests the formation of a liquid-crystalline phase (Fig. 2c). For higher concentrations over 32 vol%, phase separation of aggregated particles from the liquid-crystalline phase is observed (Fig. 2d). These results suggest that the resultant nanocrystalline calcite particles exhibited liquid-crystalline phases in the volume fraction range between 23 vol% and 32 vol% in aqueous colloidal solutions.

**Fig. 3:**
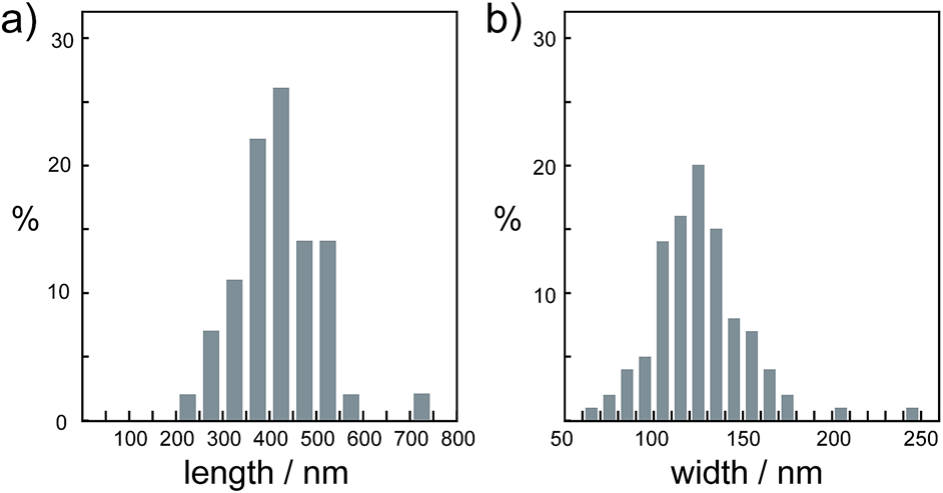
Size distribution histograms of nanorod calcite crystals obtained from the precursors formed in 7.2 × 10^–1^ wt% PAA solution for (a) length and (b) width.

**Fig. 4:**
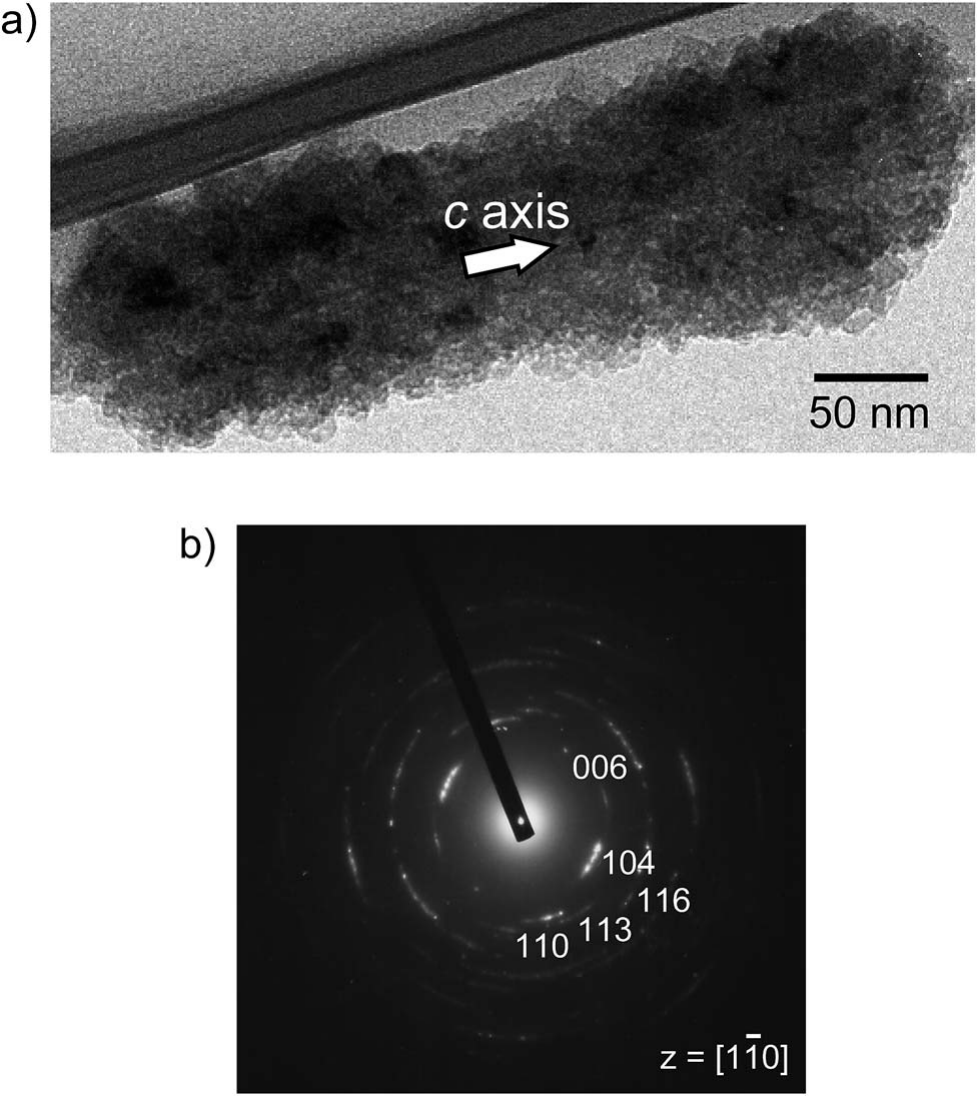
(a) TEM image and (b) SAED pattern of a calcite nanorod obtained from the precursors formed in 7.2 × 10^–1^ wt% PAA solution.

It should be noted that this colloidal liquid-crystalline phase was induced by rod-shaped calcite crystals with a relatively narrow size distribution. Fig. 3 shows the histograms obtained by measurement of the length and width of about 100 samples of CaCO_3_ nanorods pictured in SEM images. The average length and width of the CaCO_3_ nanorods were 419 ± 89 nm and 126 ± 25 nm, respectively, suggesting that the aspect ratio of the nanorods was 3.3. The crystallographic direction of the calcite nanorods was examined with a transmission electron microscope (TEM) (Fig. 4a) and selected-area electron diffraction (SAED) analysis (Fig. 4b). The TEM images show that each nanorod is not a single crystal, but is instead composed of nanocrystallites around 10–20 nm in size (Fig. 4a). For the XRD study of the crystals (Fig. S1, ESI†), the size of nanocrystals was estimated to be 15 nm according to the Scherrer equation (see the ESI†), which is in good agreement with the size of the nanocrystallites estimated based on the observations by TEM (Fig. 4a). The arched spots in the SAED pattern (Fig. 4b) show that these nanocrystallites are crystallographically oriented and their *c* axes are directed along the long axis of the nanorod. These results suggest that the nanorods exhibit a mesocrystalline structure^14^ consisting of oriented nanocrystallites around 10–20 nm in size. The mesocrystalline structure within the nanorods induced a birefringent texture in aqueous suspension (Fig. 2c).

Generally, repulsion forces between nanoparticles are necessary to inhibit aggregation of particles and to form liquid-crystalline phases in colloidal suspension.^15^ The zeta potential was measured for calcite nanorods dispersed in Na_2_CO_3_ aqueous solution (pH 10.7). The zeta potential was –55.3 ± 8.5 mV, indicating that the nanorods have a negative potential. The thermogravimetric measurement of the nanorods reveals that PAA remained and was included in the calcite nanorods (Fig. S3, ESI†). For the materials in the present study, the negatively charged PAA is adsorbed onto the surface of the calcite nanorods and generates an electrostatic repulsion force between nanorods, leading to exhibition of the liquid-crystalline phase.

**Fig. 5:**
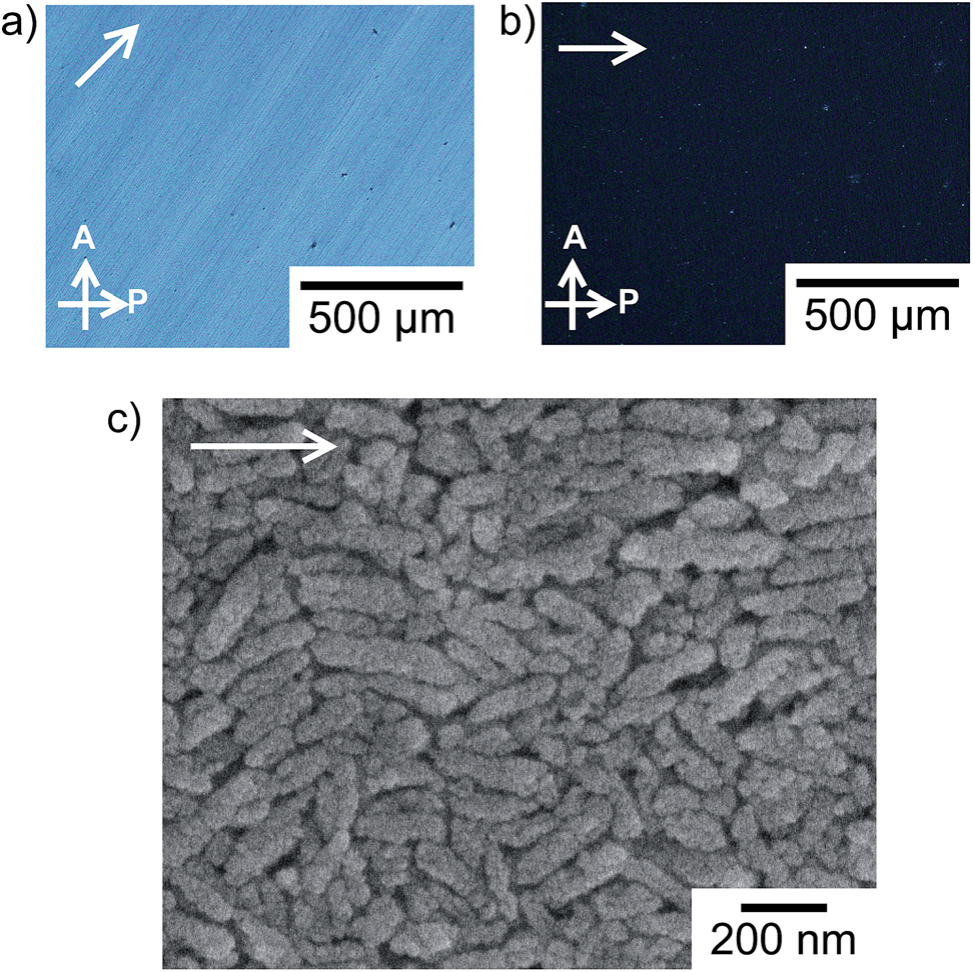
(a and b) POM images of oriented calcite nanocrystals prepared by mechanical shearing of the liquid-crystalline phase, (b) is a 45° rotation of the sample in image (a). (c) SEM image of the assembly of calcite nanocrystals oriented by mechanical shearing. The white arrows in each photograph indicate the direction of mechanical shearing.

Macroscopic liquid-crystalline alignment of the anisotropic colloidal solution with mechanical shearing is achieved for the liquid-crystalline phases including 30 vol% calcite nanorods as shown in Fig. 5. The oriented structure was maintained even after removal of water by evaporation. The image of the assembly of calcite nanorods changes from bright to dark on each rotation of the sample by 45° between crossed polarizers (Fig. 5a and b). Such behavior was observed for macroscopically-aligned thermotropic liquid crystals.^9^ The sample was also observed by POM with a tint plate (*λ* = 530 nm) inserted. When the optical axis of the tint plate was parallel to the shearing direction, a red interference colour was observed (Fig. S4a, ESI†). As the sample was rotated, the colour changed to purple and blue (Fig. S4b and S4c, ESI†). Based on the fact that calcite is optically uniaxial and negative,^16^ these results show that the *c* axes of the calcite crystals are aligned parallel to the shearing direction on a macroscopic scale and the intrinsic birefringence of each calcite nanorod due to their mesocrystalline structures largely gives rise to the birefringence in the POM image (Fig. 5a). Fig. 5c shows the SEM image of the assembly of calcite nanorods. The long axis of the calcite nanorods is overall aligned along the shearing direction (Fig. 5c), while the uniform textures are observed by POM (Fig. 5a and b). In previous studies,^17^ liquid-crystalline molecules macroscopically aligned by shearing also exhibited periodic bright and dark changes in POM observations with sample rotation, although the oriented assembly showed arched spots in the two-dimensional X-ray scattering pattern indicating disordered molecular alignment.

**Fig. 6:**
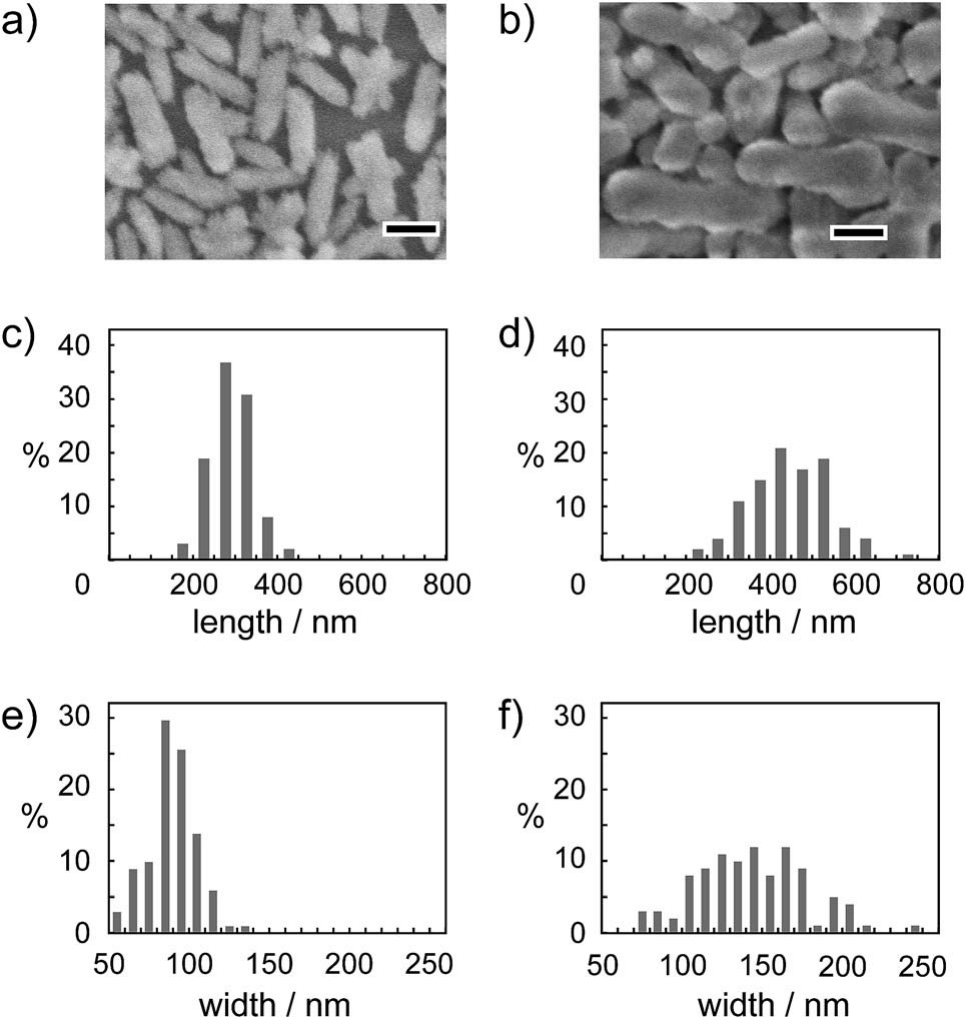
(a and b) SEM image and (c–f) size distribution of the calcite nanorod crystals formed in 10 mM (a, c, and e) and 50 mM (b, d, and f) of Na_2_CO_3_ aqueous solutions. The scale bars in (a) and (b) indicate 200 nm.

The effect of Na_2_CO_3_ concentration on the size and morphology of calcite nanorods was examined using different concentrations of Na_2_CO_3_ in aqueous solution (10–50 mM) for crystallization from the ACC precursors (Step 2 in Fig. 1). The size of the nanorods increased with increasing concentration of Na_2_CO_3_. The morphologies of the calcite nanorods were independent of the change of the concentration of Na_2_CO_3_ (Fig. 6). Table 1 shows the average particle size and aspect ratio for the calcite nanorods. On the basis of these results, the crystallization rate was less affected by the concentration of Na_2_CO_3_. These results suggest that PAA sufficiently stabilizes amorphous precursors so that the precursors are not sensitive to the salt concentration. The size of the calcite nanorods was tuned in the length range between 300 nm and 450 nm by varying the Na_2_CO_3_ amount without changing the morphology.

**Table 1:** Average particle sizes and aspect ratios for calcite nanorods formed from ACC precursors in Na_2_CO_3_ aqueous solutions with different concentrations

Na_2_CO_3_ concentration	Length (nm)	Width (nm)	Aspect ratio (length/width)
10 mM	288 ± 47	89 ± 14	3.12 ± 0.37
25 mM	419 ± 89	125 ± 26	3.35 ± 0.38
50 mM	448 ± 98	145 ± 34	3.12 ± 0.38

## Conclusions

In summary, we have demonstrated the preparation of calcite nanocrystals obtained by a self-organization process and assembled properties for colloidal liquid crystals. Through the liquid-crystalline phase, calcite crystals assemble into one-dimensionally oriented structures by mechanical shearing. Calcite nanorods have been obtained by the control of crystallization from ACC. The size can be tuned by changing the Na_2_CO_3_ concentration. The present study has demonstrated that the crystallization control inspired by biomineralization can provide CaCO_3_ nanocrystals with well-tuned structures which are preferable to induce liquid-crystalline phases. Because calcite can be used as an optical device due to its high refractive index,^18^ alignment of calcite crystals using the liquid-crystalline phases could lead to the bottom-up formation of hybrid structures exhibiting optical properties in addition to mechanical strength. CaCO_3_ is also useful for biomaterials because of its biocompatibility.^19^ CaCO_3_-based liquid-crystalline materials may have high potential in bioapplications such as bioresorbable materials,^20^ drug delivery systems^21^ and biosensors^22^ where many efforts are devoted to develop new materials based on liquid-crystalline states. Further investigation of the morphological control of CaCO_3_ crystals could lead to a variety of assembled structures and the development of novel functional environmentally friendly materials.

## Experimental section

### Preparation of the CaCO_3_ colloidal solution

The typical procedure for the preparation of colloidal CaCO_3_ crystals is as follows. The amorphous calcium carbonate (ACC) precursor^13^ was prepared by mixing of 20 mL of 100 mM CaCl_2_ aqueous solution including PAA (*M*_w_ = 2.0 × 10^3^, 7.2 × 10^–1^ wt%) and an equal volume of 100 mM Na_2_CO_3_ aqueous solution. This solution was stirred for 1 h at room temperature, and the precipitates were then centrifuged. The precipitates were re-dispersed without drying in 40 mL of 25 mM Na_2_CO_3_ aqueous solution. The reaction solution was stirred for 72 h at room temperature to crystallize ACC. After centrifugation of the suspension, the supernatant was decanted to condense CaCO_3_ crystals.

### Characterization

X-ray diffraction (XRD) patterns were recorded with a SmartLab (Rigaku) diffractometer with Cu Kα radiation in steps of 0.02° over the range from 20 to 60°. Fourier transform infrared (FTIR) spectra were recorded on a Jasco FT/IR-6100 spectrometer (Jasco, Tokyo) with the KBr method. Thermogravimetric measurements (Rigaku, TG-8120) were conducted up to 1000 °C under N_2_ flow conditions (100 mL min^–1^). Optical properties of the samples were observed with a polarizing optical microscope (Olympus, BX51). The morphologies of the crystals were observed with scanning electron microscopy (SEM) (Hitachi, S-4700) and transmission electron microscopy (TEM) (JEOL, JEM-2010HC, operated at 200 kV). The zeta potential was measured at 25 °C by using a Zetasizer (Nano-ZS, from Malvern Instruments Ltd) for the colloidal solution of the nanorods (pH 10.7) obtained from ACC precursors dispersed in 25 mM Na_2_CO_3_ aqueous solution (Step 2 in Fig. 1).

### Materials

All chemical reagents used for syntheses of CaCO_3_ crystals were obtained from commercial sources. PAA (*M*_w_ = 2.0 × 10^3^) was purchased from Sigma-Aldrich. CaCl_2_ was obtained from Wako. Na_2_CO_3_ was obtained from Kanto Chemical. All reagents were used without purification. Deionized water, obtained by using an Auto Pure WT100 purification system (Yamato), was employed as the solvent for syntheses of CaCO_3_ crystals.

## Supplementary Material

Supplementary information
