# Supplementary material for: Liquid-crystalline calcium carbonate: biomimetic synthesis and alignment of nanorod calcite
Source: Chem Sci. 2015 Aug 12;6(11):6230–4. doi: 10.1039/c5sc01820j (PMC6054116; doi:10.1039/c5sc01820j)
Supplement: Supplementary file 1 [file SC-006-C5SC01820J-s001.pdf]

Electronic Supplementary Material (ESI) for Chemical Science.
This journal is © The Royal Society of Chemistry 2015

Electronic Supplementary Information for

Liquid-crystalline calcium carbonate: biomimetic synthesis and alignment of nanorod calcite

Masanari Nakayama,[a] Satoshi Kajiyama,[a‡] Tatsuya Nishimura,[a] and Takashi Kato[a]*

[a] *Department of Chemistry and Biotechnology, School of Engineering, The University of Tokyo, Hongo, Bunkyo-ku, Tokyo 113-8656, Japan*

‡ *Current address: Department of Chemical System Engineering, School of Engineering, The University of Tokyo, Hongo, Bunkyo-ku, Tokyo 113-8656, Japan*

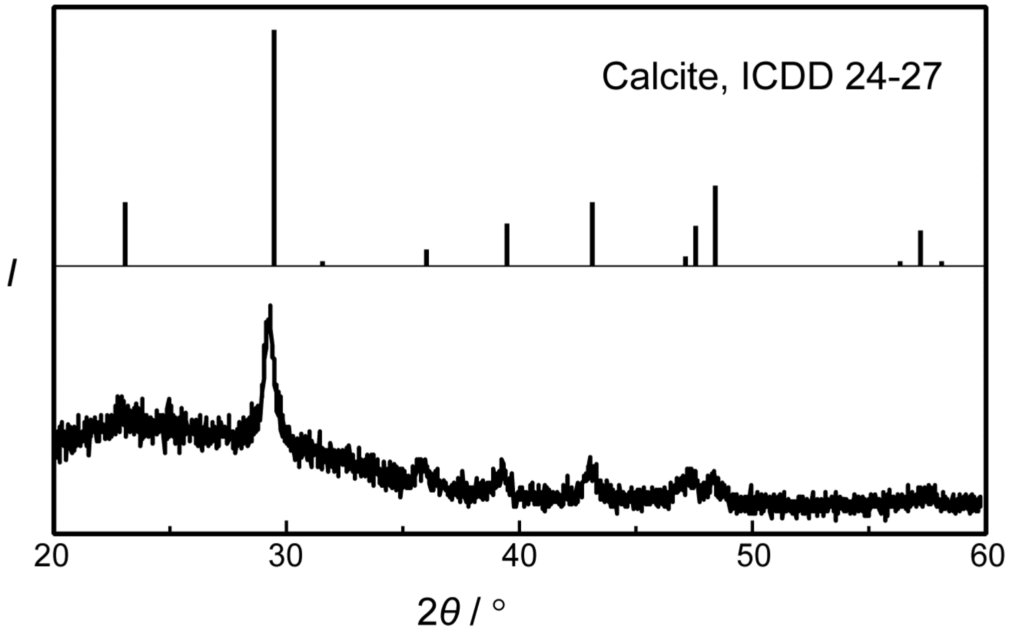

Figure S1. X-ray diffraction (XRD) pattern for the $CaCO_3$ crystals obtained from the precursors formed in 7.2×10^{-1} wt% PAA solution.

Characterization of the crystalline phase of the nanocrystals

All peaks in the XRD pattern (Figure S1) are characteristic for calcite.

The particle size estimation with the Scherrer equation

The broad peaks in the XRD pattern (Figure S1) suggest the nanocrystalline structure of obtained $CaCO_3$ crystals. We estimated the primary particle size from the line width of (104) peak with the Scherrer equation,

$$\tau = \frac{K\lambda}{\beta \cos \theta}$$

where τ is the particle size, β is full width at half maximum of the peak, θ is Bragg angle, K is shape factor (for isotropic particle, K is 0.90), and λ is wavelength of X-ray (1.5418 Å, CuKα). The XRD pattern in Figure S1 shows the line width β of (104) peak (θ= 14.73°) is 0.52. If the primary particle is presumed to be isotropic, the size is estimated to be 15 nm.

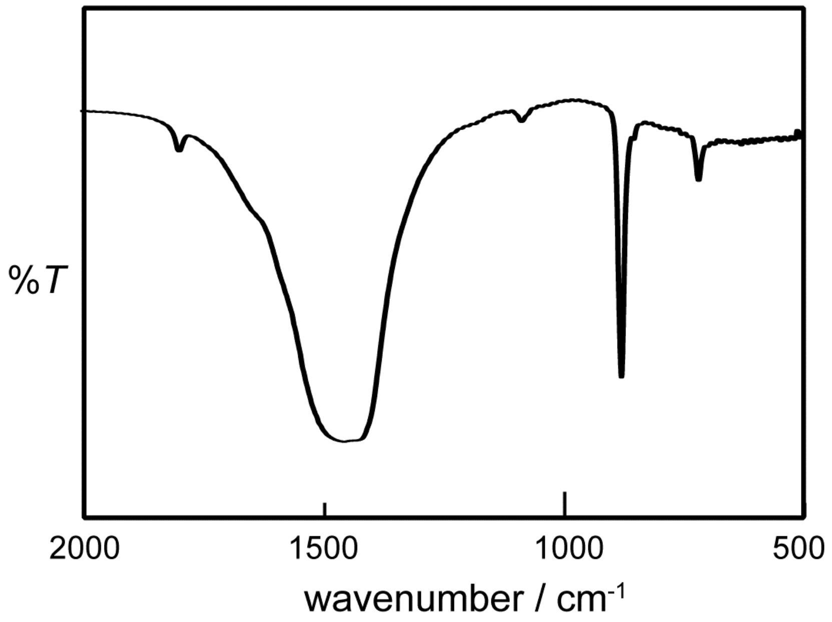

Figure S2. Fourier-transform infrared (FTIR) spectrum for the $CaCO_3$ crystals obtained from the precursors formed in 7.2×10^{-1} wt% PAA solution.

Characterization of the crystalline phase of the nanocrystals

The peaks at 873 cm^{-1} and 714 cm^{-1} (Figure S2) are attributed to the vibration of out-of-plane bending (v_2) and in-plane (v_4) bending of CO_3^{2-} in calcite, respectively. No peak attributable to the other $CaCO_3$ polymorphs is observed.

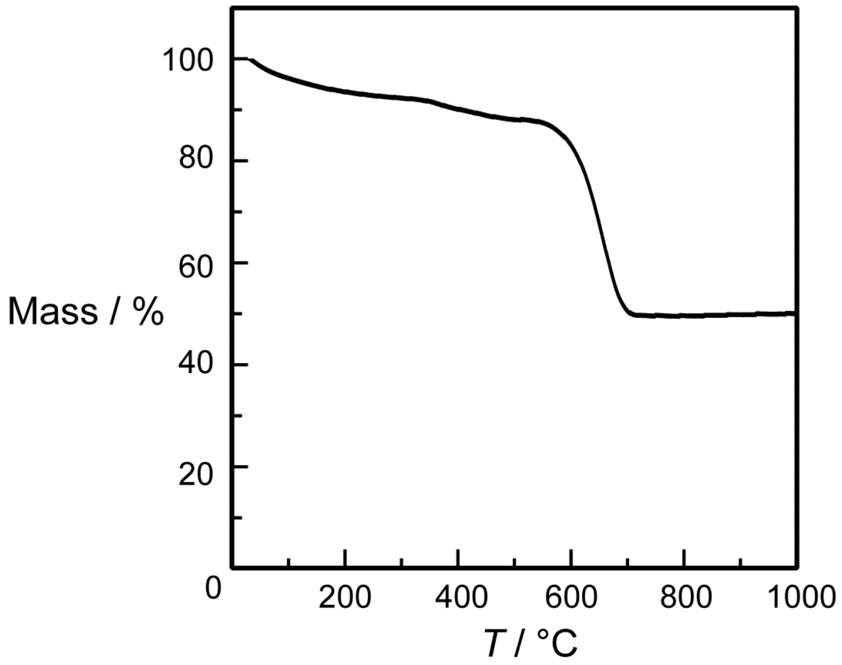

Figure S3. Thermogravimetric curve of the precipitates obtained after drying of the colloidal liquid-crystalline suspension under vacuum. The thermogravimetric curve was recorded to 1000 °C at heating rate of 5 °C/min under a N_2 flow condition.

Composition analysis of the calcite nanocrystals

The weight loss attributable to water molecules is observed below 200 °C (Figure S3). PAA decomposes from 200 °C to 600 °C. The weight loss of CO_2 from $CaCO_3$ crystals is seen over 600 °C. The composition of the nanorods was calculated to be 6 wt% water molecules, 7 wt% PAA and 87 wt% calcite crystals.

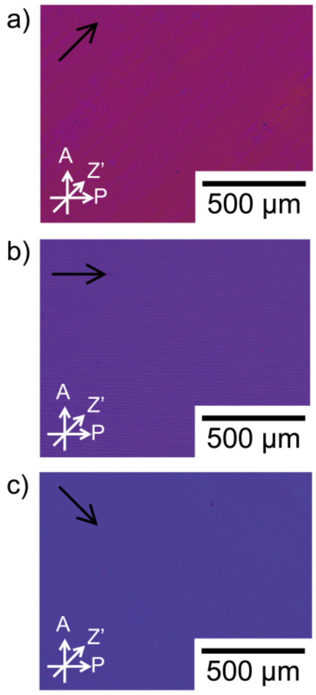

Figure S4. Polarizing optical microscope (POM) images with a tint plate (λ = 530 nm) of calcite nanorods aligned by mechanical shearing. The black arrows in each photograph indicate the direction of mechanical shearing.

Observation for the oriented direction of *c* axes of the calcite nanocrystals

The interference color changes from red (Figure S4a) to purple (Figure S4b) and blue (Figure S4c) as the sample is rotated, indicating that the *c* axes of the assembled calcite crystals are aligned parallel to the shearing direction.
